# The Role of Equanimity in Predicting the Mental Well-Being of the Residents in Long-Term Care Facilities in Thailand

**DOI:** 10.3390/nursrep15040123

**Published:** 2025-04-03

**Authors:** J J Maung, Justin DeMaranville, Tinakon Wongpakaran, Carmelle Peisah, Suthikarn Arunrasameesopa, Nahathai Wongpakaran

**Affiliations:** 1 Mental Health Program, Multidisciplinary Interdisciplinary School, Chiang Mai University, Chiang Mai 50200, Thailand; jjmaung_jj@cmu.ac.th (J.J.M.); justinross.dem@cmu.ac.th (J.D.); tinakon.w@cmu.ac.th (T.W.); c.peisah@unsw.edu.au (C.P.); 2 Department of Psychiatry, Faculty of Medicine, Chiang Mai University, Chiang Mai 50200, Thailand; 3 Discipline of Psychiatry and Mental Health, Faculty of Medicine and Health, University of New South Wales, Sydney, NSW 2052, Australia; 4 Specialty of Psychiatry, Faculty of Medicine and Health, University of Sydney, Sydney, NSW 2006, Australia; 5 Panyananthaphikkhu Chonprathan Medical Center, Srinakharinwirot University, Nonthaburi 11120, Thailand; stamp.suthikarn@gmail.com

**Keywords:** equanimity, mental well-being, older people, long-term care facilities, nursing

## Abstract

**Background/Objectives**: The prevalence of mental health issues, such as depression, loneliness, and a decreased quality of life among older adults in long-term care (LTC) facilities in Thailand, calls for further study. In Thailand, where Buddhism is the predominant religion, many positive psychological strengths are fostered among older adults. One notable strength is equanimity, which is characterized by a balanced and accepting response to both positive and negative events. This practice is commonly associated with enhancing the well-being of older individuals. However, the study between equanimity and well-being is scarce. The purpose of the study is to assess equanimity in LTC residents and to determine if it is a significant predictor of the mental well-being of the residents. **Methods**: The cross-sectional data was obtained from 236 LTC residents in Thailand. Equanimity was measured using the Inner Strength-Based Inventory (iSBI) and mental well-being from the Thai Geriatric Depression Scale (TGDS-6). Demographic factors, depression, loneliness, and other inner strengths were also explored as covariates in a logistic-regression analysis. **Results**: The mean scores for equanimity (Mean = 3.78 ± 1.00) and mental well-being (Mean = 0.720 ± 0.449) were determined. The multiple regression analysis found equanimity significantly predicted well-being (B = 0.593, *p* = 0.002) along with depression (B = −0.395, *p* < 0.001) and mindfulness (B = 0.355, *p* = 0.046). **Conclusions**: This study identifies equanimity as a key predictor of mental well-being among Thai long-term care residents, alongside depression and mindfulness. While the study’s cross-sectional design limits causal conclusions, the results suggest that incorporating equanimity-based practices into geriatric care could be beneficial. Future longitudinal research is needed to validate these findings and improve resilience and well-being in aging populations.

## 1. Introduction

Global aging is a pressing issue, with the number of older adults projected to double to over 1 billion by 2050 [1]. Thailand, which became an aging society in 2005, is experiencing this trend acutely. In 2018, 11.8 million people (17.8% of the population) were aged 60 and over, and this figure is projected to exceed 30% by 2050, transitioning Thailand into a super-aged society [2]. This demographic shift, driven by increased life expectancy and declining birth and death rates, has broken traditional extended family structures, resulting in fewer caregivers for older adults and a growing reliance on long-term care (LTC) facilities [3,4,5]. The mental health of LTC residents is a significant concern, with depression rates ranging from 5% to 41%, some of the highest rates reported in Thailand [5]. Depression in this population is associated with loneliness, lower quality of life, and increased risks of cognitive decline, cardiovascular disease, and even suicide [6,7]. A key discipline in this effort is psychiatric and mental health nursing, which focuses on community-based, holistic, and person-centered approaches to promote quality of life for the older population. Nurses play an essential role in such a way by incorporating holistic and psychological approaches into routine care [8]. Addressing these challenges requires innovative approaches beyond traditional medical care, focusing on holistic and person-centered interventions to promote mental well-being and quality of life [9].

Mental well-being, a multidimensional construct encompassing happiness, life satisfaction, and emotional health, is critical for successful aging [10]. Positive mental well-being has been shown to reduce the risk of mental illnesses such as anxiety and depression, making it a key target for interventions in older populations [10,11,12]. One promising yet under-explored factor in promoting mental well-being is equanimity, a psychological trait characterized by emotional balance, tolerance, and a non-judgmental attitude toward oneself and others [13,14,15]. Equanimity is deeply rooted in Thai culture, particularly among older adults, as it is a fundamental aspect of the Brahma-viharas (the Four Immeasurables) in Theravada Buddhism, the dominant religion in Thailand. The Brahma-viharas, which include loving-kindness (Metta), compassion (Karuna), empathetic joy (Mudita), and equanimity (Uppekha), are considered essential moral qualities for spiritual development [16,17]. These qualities are not only central to Buddhist teachings but are also actively cultivated through meditation and mindfulness practices, which are widely practiced in Thailand. In Thai culture, older adults often engage in meditation to cultivate these virtues, particularly equanimity, in their daily lives. This practice not only promotes emotional health but also enhances one’s ability to cope with the inevitable challenges associated with aging, such as health issues, loss of loved ones, or changes in living arrangements [18]. Furthermore, equanimity is one of the ten perfections in Buddhism, which are inner strengths cultivated for spiritual growth and enlightenment. The ten perfections include qualities such as generosity, morality, patience, and equanimity, with the latter being particularly emphasized for older adults as they navigate the later stages of life [17].

In Thai culture, older adults are often seen as spiritual role models, and equanimity is encouraged to achieve inner peace and resilience in the face of life’s challenges [19]. This cultural and spiritual context makes equanimity a highly relevant and accessible resource for promoting mental well-being among older adults in Thailand, particularly those residing in LTC facilities, where emotional distress and loneliness are prevalent [20]. The LTC setting presents unique challenges that make equanimity a particularly relevant and valuable construct to study. Residents in LTC facilities often face significant life transitions, such as the loss of independence, separation from family, and adaptation to institutional living, which can lead to feelings of helplessness, loneliness, and emotional distress [21]. These challenges are compounded by the high prevalence of depression and cognitive decline in this population, creating a pressing need for interventions that address both emotional and psychological well-being [22,23].

Equanimity, emphasizing emotional balance and acceptance, offers a promising framework for addressing these challenges. By fostering a non-judgmental and tolerant attitude toward life’s difficulties, equanimity can help LTC residents cope with the stressors of institutional living, such as loss of autonomy and limited social interactions. For example, equanimity can enable residents to accept their circumstances without excessive emotional reactivity, reducing feelings of frustration and helplessness [24]. Additionally, equanimity can enhance resilience, allowing residents to maintain a sense of inner peace and stability despite external challenges [15]. Moreover, equanimity aligns well with the cultural and spiritual values of older Thai adults who have practiced meditation and mindfulness throughout their lives. This cultural familiarity makes equanimity-based interventions more likely to be accepted and effectively implemented in LTC settings [25]. For instance, mindfulness practices, such as meditation on the Brahma-viharas, can be integrated into daily routines to promote emotional regulation and mental well-being [26]. Given the high rates of depression and loneliness among LTC residents, equanimity-based approaches could serve as a culturally appropriate and cost-effective way to improve mental health outcomes in this vulnerable population [15,27]. While equanimity has been studied in various contexts, such as mindfulness and workplace stress, its application in Long-Term Care (LTC) settings, particularly in Thailand, remains underexplored. Existing research suggests that equanimity can buffer against adverse psychological outcomes, such as neuroticism and perceived stress, and is positively associated with subjective health and well-being in older adults [13,14]. For example, a study on the Thai population found that high levels of equanimity mitigated the effects of neuroticism and perceived stress on depressive symptoms, highlighting its potential as a protective factor for mental health [13]. Another study found that the acceptance aspect of equanimity was positively correlated with subjective health and well-being in older adults, further underscoring its relevance for this population [28].

However, there is a significant gap in understanding how equanimity influences the mental well-being of LTC residents, a population particularly vulnerable to emotional distress and loneliness. This study aims to address this gap by investigating the levels of equanimity among LTC residents in Thailand and examining its role in predicting mental well-being. By doing so, it seeks to provide evidence for the potential of equanimity-based interventions in LTC settings, offering a novel approach to improving older adults’ quality of life and emotional health. Given the scarcity of research on equanimity in LTC populations, this study has the potential to contribute valuable insights to both the scientific community and healthcare practitioners, paving the way for innovative, holistic care strategies tailored to the unique needs of this vulnerable group.

## 2. Methods

### 2.1. Setting and Design

This was a secondary analysis of the study “Influence of Attachment Anxiety on the Relationship between Loneliness and Depression among Long-Term Care Residents” [5]. The original data was collected from 12 government long-term care (LTC) homes across Thailand between December 2020 and July 2021. The participants were LTC residents aged 60 or older who met specific inclusion criteria, such as being able to communicate orally in Thai and complete questionnaires independently. Exclusion criteria included conditions like dementia, significant sensory impairments, or a Mini-Cog score of less than 3 points. This indicates that the original data collection was focused on understanding psychological and social factors (e.g., attachment anxiety, loneliness, and depression) among elderly LTC residents.

The original study aimed to explore the relationship between attachment anxiety, loneliness, and depression among LTC residents. The secondary analysis extends this by examining equanimity, an inner strength that was included in the original data but not analyzed. While the original study focused on psychological distress (e.g., depression and loneliness), the secondary analysis shifts focus to a protective factor (equanimity), which may not have been a primary objective of the original study. However, the data collected (e.g., psychological and social variables) appears relevant to both studies.

The data was collected through questionnaires administered to LTC residents who met the inclusion criteria. The methods of data collection (e.g., self-reported questionnaires) are consistent with the objectives of both the original and secondary studies, as both focus on psychological and social constructs. The secondary analysis introduces a new variable (equanimity) that was not analyzed in the original study. This suggests that the secondary analysis involves different statistical approaches or models to explore the relationship between equanimity and other variables (e.g., loneliness and depression).

LTC homes, managed by the Ministry of Social Development and Human Security in Thailand, accommodate elderly individuals who apply for residency. In Thai culture, older adults generally live with their children. However, those who do not have family or who have been abandoned require appropriate care. Such individuals are eligible for residency in LTC homes. The long-term care (LTC) homes in Thailand have about 40 percent of their residents diagnosed with dementia. These residents often stay for the remainder of their lives. The typical size of these homes ranges from 100 to 300 residents [29].

### 2.2. Sampling

G-Power was used to calculate the sample size required for the regression analysis, which was 194 participants at minimum. The effect size was 0.15, the alpha error probability was 0.05, and the power was 0.95. The total sample acquired from the secondary data sample was N = 236.

Inclusion criteria—(1) LTC older residents who were 60 years and older, (2) LTC older residents who were proficient in communicating in Thai, and (3) LTC older residents who could independently complete the questionnaires.

Exclusion criteria—(1) Residents with significant weakness that interfered with research participation (e.g., visual impairment preventing test completion), and (2) Residents diagnosed with dementia or a Mini-Cog score of less than three.

### 2.3. Measure of Equanimity

One of the key teachings of Theravada Buddhism includes the 10 perfections or *Paramis*, which are seen as favorable psychological characteristics [30]. These characteristics show a resemblance to the description of Seligman and Csikszentmihalyi’s character strengths [30,31]. They represent the core positive qualities of the self that drive psychological growth and adaptation and are regarded as “inner strengths”. The inner Strength-Based Inventory (iSBI) focuses on these *Paramis* where equanimity is included as one of the factors along with generosity, morality, mindfulness, wisdom, perseverance, endurance, truthfulness, determination, and loving-kindness. The measure of equanimity is taken from the iSBI where the scale consists of the question “When I encounter losses/separations”, the five choices of response include varying degrees of how easily a person can recover from losses or separations. Regarding its psychometric property, iSBI demonstrated that the person separation was 2.45 for iSBI, corresponding to good reliability coefficients of 0.86. Item reliability was 0.99 (>0.80), and Cronbach’s alpha was 0.71 in older adults. The individual items in iSBI demonstrated unidimensionality and local independence in the previous psychometric study, allowing each item to be measured independently. Good reliability was exhibited with the interclass correlation coefficient of the Equanimity scale of 0.88 (95% CI = 0.70, 0.95, *p* < 0.0001) from the two-week test-retest reliability in the Thai general population. Though the iSBI has never been tested in the LTC setting, it demonstrated that Cronbach’s alpha for the LTC residents was 0.79.

### 2.4. Measure of Mental Well-Being

Mental well-being entails more than not having a mental illness; it is a broader concept including, but not limited to, positive emotions such as happiness, contentment, satisfaction, and enjoyment [32,33,34]. The items addressing the subjective experience of mental well-being, drawn from the Thai Geriatric Depression Scale (TGDS-6), included “Are you basically satisfied with your life?” and “Are you in good spirits most of the time?” These two items infer life satisfaction and good mood congruent with the subjective well-being concepts of Diener et al. [33] and the WHO-5 well-being index [35].

### 2.5. Data Analysis

Descriptive analysis for age, sex, education, and marital status was provided as percentages, averages, and standard deviations. Bivariate correlation analysis employing Pearson’s correlation methods was performed to explore correlations among variables.

Logistic Regression Analysis was applied to determine whether equanimity is a significant predictor of the mental well-being of older residents in the LTC setting. Loneliness and Depression were treated as covariates and were accounted for, along with other inner strengths and demographic characteristics.

The logistic regression model was specified as follows:

log(*p*/1 − *p*) = β0 + β1(Equanimity) + β2(Loneliness) + β3(Depression) + β4(Truthfulness) + β5(Perseverance) + β6(Wisdom) + β7(Generosity) + β8(Morality) + β9(Mindfulness) + β10(Patience and Endurance) + β11(Determination) + β12(Loving-Kindness) + β13(Demographic Characteristics);

where *p* represents the probability of high mental well-being, β0 is the intercept, and β1 to β13 are the regression coefficients for the predictor and covariates.

Depression was investigated through the TGDS-6, and Loneliness was measured by a 6-item revised version of the UCLA Loneliness Scale (RULS-6). The latter consists of six options, multiple-choice, with a score from 1 to 4, with a total score ranging from 6 to 24. The higher the score, the higher the level of loneliness. Cronbach’s alpha of RULS-6 was 0.83 [36]. SPSS software (IBM SPSS Version 27) was used for analysis. If the 95% confidence interval (CI) excludes the null value, the null hypothesis is rejected, and a *p*-value below 0.05 is considered statistically significant.

## 3. Results

### 3.1. Demographic Information

Demographic data are reported in Table 1.

### 3.2. Equanimity and Mental Well-Being

Table 2 presents the frequency, percentage, and mean of the variables equanimity (i-SBI Equanimity) and mental well-being (TGDS).

### 3.3. Correlation of Variables

Table 3 describes the correlation between variables including age, sex, education, marital status, equanimity, mental well-being, loneliness, depression, and the rest of the inner strengths—truthfulness, perseverance, wisdom, generosity, morality, mindfulness, patience (endurance), determination, and loving-kindness.

Equanimity is significantly correlated with higher well-being (*r* = 0.297, *p* < 0.01) and is negatively associated with depression (*r* = −0.160, *p* < 0.05). Furthermore, equanimity significantly correlates with the rest of all the inner strengths. As for depression, it shows a negative association with well-being (*r* = −0.258, *p* < 0.01) and a positive association with loneliness (*r* = 0.347, *p* < 0.01).

### 3.4. Multiple Logistic Regression of Variables

Table 4 shows the multiple logistic regression across 1000 bootstrap samples generated from the original dataset, applied to evaluate the association between well-being and the predictor variable, equanimity, and other covariables.

## 4. Discussion

The objectives of this study were to assess equanimity in LTC residents and to determine if equanimity was a predictor of mental well-being. The results suggest that equanimity significantly predicted the mental well-being of LTC residents, as hypothesized. Equanimity was strongly linked to mental well-being even after controlling for other inner strengths and covariates, such as depression and loneliness, suggesting that equanimity represents a unique role in the mental well-being of LTC residents, independent of the influence of other factors. As far as we know, this has hitherto been unexplored in LTC.

These findings coincide with previous studies that highlight the role of equanimity as a positive psychological characteristic that promotes emotional regulation and resilience [13,14,15]. Additionally, the findings also support the existing evidence that links depression and mindfulness to psychological well-being, where mindfulness positively influences well-being and depression negatively does so, indicating their importance as determinants of mental health [5,10].

The mean score of equanimity for this population is comparable to that reported in diverse Thai populations, including medical students [30] and the general participants [13]. This indicates that the participants in this study could recover from setbacks related to losses or separations at a rate comparable to those around them by mitigating the negative emotions and balancing reactions in favor of a more neutral response. This ability to limit the severity of one’s reaction to setbacks highlights one potential mechanism of equanimity: reduced reactivity to adversities and increased tolerance to distress [37,38]. The negative relationship between equanimity and depression in this study’s sample highlights how a balanced response to loss may mitigate depressive symptoms. Resilience, which implies adaptability and flexibility to difficulties and challenges [39], shows resemblance with equanimity; however, the question “When I encounter losses/separations” was applied to measure equanimity because this scale helps to measure how well someone can stay calm, composed, and emotionally balanced during life’s challenges, which is a core aspect of equanimity [13,15,37], imposing a broader construct that is more than the ability to adapt and be flexible.

This study is not without its limitations. Firstly, cross-sectional design limits the ability to establish causality of the link between equanimity and mental well-being. Secondly, generalizability is limited because the sample is derived from abandoned older persons living in LTC facilities under the Ministry of Social Development and Human Security, which may not represent older individuals in alternative environments or those residing independently. Thirdly, the primary data was collected during the COVID-19 outbreak, which may have influenced the results of the study [5,14,40]. Fourthly, applying a single-item question to measure equanimity may not fully encompass the psychological construct of equanimity. Finally, this study did not include other potential external factors such as length of stay, physical illnesses, and traumatic life experiences.

One limitation of this study is the exclusion of individuals with a diagnosis of dementia, which may restrict the generalizability of our findings to the broader LTC population. In many regions, including Europe, a considerable proportion of LTC residents have dementia, and their exclusion may overlook important insights into the role of equanimity in this vulnerable group. Future research should explore adapted methodologies to include individuals with dementia, such as using proxy measures (e.g., caregiver-reported equanimity or behavioral observations) or employing simplified assessment tools designed for individuals with cognitive impairments. Additionally, qualitative approaches, such as interviews with caregivers or family members, could provide valuable perspectives on equanimity in this population. By addressing these methodological challenges, future studies can ensure a more inclusive understanding of equanimity and its impact on mental well-being across diverse LTC populations.

### 4.1. Clinical Implications for Nurses

Nurses in long-term care (LTC) facilities play a pivotal role in supporting residents’ mental well-being by integrating holistic and psychological approaches into routine care [8]. Equanimity-based interventions or combining equanimity with other interventions, such as emotional and physical experiences and labeling sensations, could serve as protective psychological resources for aging populations [41]. Older adults often face significant life transitions, including social losses, physical decline, and heightened vulnerability to depression and anxiety [8]. Nurses can help residents navigate these challenges and enhance their mental health outcomes by fostering emotional balance, purpose, and adaptive resilience.

### 4.2. Suggestions for Future Research

Despite this study’s novelty in addressing gaps in equanimity research, further investigation is needed to advance both theory and practice. Future studies should (1) test equanimity-based interventions in LTC settings to evaluate their efficacy in reducing depression, anxiety, and loneliness among residents, (2) explore cultural adaptations of equanimity training, ensuring relevance to diverse populations and caregiving contexts, (3) conduct longitudinal or experimental designs to establish causal links between equanimity, inner strengths, and mental health outcomes, (4) investigate how equanimity interacts with other psychological constructs (e.g., resilience, mindfulness) to inform integrated care models.

## 5. Conclusions

This study highlights the significance of equanimity as a crucial predictor of mental well-being among residents in long-term care (LTC) facilities in Thailand, alongside other factors such as depression and mindfulness. With a rapidly aging population, promoting equanimity through targeted interventions may help address mental health challenges.

Given the global rise in aging populations and the increasing dependence on long-term care facilities, these findings could guide public health strategies aimed at improving the mental well-being of LTC residents. When integrated into community-based mental health care and LTC nursing programs, methods focusing on equanimity could provide practical tools to enhance emotional well-being and overall quality of life. Future research should utilize longitudinal study designs to explore the long-term relationship between equanimity and mental well-being. Additionally, it would be valuable to investigate interventions like mindfulness-based therapies that incorporate equanimity.

## Figures and Tables

**Table 1:** Demographic characteristics of the participants (*n* = 236).

Variables	*n*	*%*
Age: mean ± SD	73.52 ± 7.32	
Sex		
Male	100	42.4
Female	136	57.6
Marital Status		
Single	109	46.2
Not Single	127	53.8
Education		
Primary or No Education	143	60.6
High School or Advanced	93	39.4

**Table 2:** Equanimity and Mental Well-being (*n* = 236).

Instruments	*n* (%)	Mean ± SD
i-SBI Equanimity (1–5): When I encounter losses/separations…		3.78 ± 1.0
Normally, I cannot get over them. I once tried to kill myself or was brought to the hospital	8 (3.4)	
It is very difficult for me to get over them, resulting in physical and mental symptoms but not so serious that I need to go to hospitals	23 (9.8)	
It is difficult and takes a long time for me to get over them	32 (13.6)	
It takes some time for me to get over them but no later than others.	121 (51.5)	
I can get over them in sooner time compared with others	51 (21.7)	
Well-being (TGDS)		0.72 ± 0.44
High (2)	170 (72.0)	
Low (0–1)	66 (28.0)	

**Table 3:** Correlation matrix of the variables.

Items	1	2	3	4	5	6	7	8	9	10	11	12	13	14	15	16	17
1. Age	--																
2. Sex (Female)	0.270 **	--															
3. Education (Higher)	0.018	−0.063	--														
4. Marital Status (Non-single)	0.092	0.031	−0.105	--													
5. Equanimity	0.033	0.094	0.109	0.068	--												
6. Well-being (Higher)	0.118	0.134 *	0.116	0.123	0.297 **	--											
7. Loneliness	0.105	0.005	−0.029	0.088	−0.087	−0.071	--										
8. Depression	−0.053	−0.172 **	−0.139 *	0.073	−0.160 *	−0.258 **	0.347 **	--									
9. Truthfulness	0.064	0.233 **	−0.085	0.058	0.155 *	0.191 **	0.032	−0.049	--								
10. Perseverance	0.042	0.113	−0.043	−0.030	0.262 **	0.214 **	−0.037	−0.028	0.333 **	--							
11. Wisdom	−0.002	00.082	0.033	0.020	0.259 **	0.208 **	−0.067	−0.142 *	0.312 **	0.423 **	--						
12. Generosity	00.044	0.210 **	0.079	0.058	0.297 **	0.220 **	−0.201 **	−0.087	0.240 **	0.409 **	0.374 **	--					
13. Morality	0.157 *	0.120	0.074	0.039	0.282 **	0.158 *	−0.145 *	−0.134 *	0.278 **	0.209 **	0.344 **	0.278 **	--				
14. Mindfulness	0.064	0.064	−0.020	00.061	0.191 **	0.214 **	−0.113	−0.050	0.169 **	0.364 **	0.345 **	0.327 **	0.381 **	--			
15. Patience and Endurance	−0.064	−0.029	0.042	0.032	0.331 **	0.066	−0.038	00.045	00.023	0.334 **	0.282 **	0.205 **	0.167 *	0.362 **	--		
16. Determination	−0.055	−0.055	0.107	0.017	0.223 **	00.082	−00.041	00.010	0.271 **	0.275 **	0.346 **	0.233 **	0.323 **	0.375 **	0.261 **	--	
17. Loving-Kindness	0.099	0.149 *	0.061	0.046	0.326 **	0.119	−0.066	−0.052	0.168 **	0.274 **	0.192 **	0.237 **	0.337 **	0.282 **	0.241 **	0.227 **	--

* p < 0.05, ** *p* < 0.01.

**Table 4:** Multiple logistic regression of predictors of well-being.

Predictor	B	SE	*p*-Value	95% LL-CI	95% UL-CI
Gender	−0.124	0.439	0.743	−0.983	0.725
Age	0.030	0.031	0.261	−0.025	0.099
Education	−0.688	0.444	0.084	−1.677	0.091
Marital Status	−0.637	0.400	0.078	−1.504	0.084
Truthfulness	0.229	0.173	0.137	−0.071	0.612
Perseverance	0.233	0.246	0.275	−0.218	0.782
Wisdom	0.063	0.190	0.702	−0.293	0.460
Generosity	0.114	0.225	0.544	−0.342	0.567
Morality	−0.086	0.206	0.645	−0.495	0.330
Mindfulness	0.355	0.196	0.046	0.000	0.809
Patience and Endurance	−0.240	0.209	0.180	−0.698	0.117
Equanimity	0.593	0.222	0.002	0.252	1.154
Determination	−0.156	0.187	0.353	−0.562	0.201
Loving-Kindness	−0.104	0.190	0.558	−0.510	0.226
Loneliness	0.008	0.051	0.847	−0.092	0.112
Depression	−0.395	0.153	<0.001	−0.753	−0.129

B = unstandardized coefficient, SE = standard error, LL = lower level, UL = Upper level, CI = confidence interval.

## Data Availability

According to the policy implemented during this study, the ethics committee does not permit the authors to share the data with other entities. The data sets used and/or analyzed for the current study are available from the corresponding author upon reasonable request.

## Public Involvement Statement

There was no public involvement in any aspect of this research.

## Guidelines and Standards Statement

This manuscript was drafted in accordance with the Strengthening the Reporting of Observational Studies in Epidemiology (STROBE) guidelines for cross-sectional studies.

## Abbreviations

The following abbreviations are used in this manuscript:

LTC	Long-Term Care
MDD	Major Depressive Disorder
iSBI	inner Strength-Based Inventory
TGDS-6	6-item Thai Geriatric Depression Scale
RULS-6	6-item Revised UCLS Loneliness Scale
